# Effect of radiochemotherapy on the cognitive function and diffusion tensor and perfusion weighted imaging for high-grade gliomas: A prospective study

**DOI:** 10.1038/s41598-019-42321-8

**Published:** 2019-04-12

**Authors:** Yiying Bian, Li Meng, Jianghua Peng, Junfeng Li, Rui Wei, Lei Huo, Huan Yang, Ying Wang, Jun Fu, Liangfang Shen, Jidong Hong

**Affiliations:** 1 Departments of Oncology, Xiangya Hospital, Central South University, Changsha, 410008 Hunan P.R. China; 2 Departments of Radiology, Xiangya Hospital, Central South University, Changsha, 410008 Hunan P.R. China; 3 Departments of Neurosurgery, Xiangya Hospital, Central South University, Changsha, 410008 Hunan P.R. China; 4 Departments of Neurology, Xiangya Hospital, Central South University, Changsha, 410008 Hunan P.R. China; 5 Departments of Pathology, Xiangya Hospital, Central South University, Changsha, 410008 Hunan P.R. China

## Abstract

This study aimed to explore the effects of radiochemotherapy on the neurocognitive function of patients with high-grade gliomas (HGG). The mini-mental state examination (MMSE), Montreal Cognitive Assessment (MoCA), event-related potential P300 (ERP-P300), and specific MRI parameters were compared, and the associations between specific MRI parameters and different doses of radiation were determined for before and up to 12 months after radiotherapy. There were no significant differences in MMSE, MoCA, or ERP-P300 before and after radiotherapy. Compared with pre-radiochemotherapy, fractional anisotropy (FA) in the contralateral hippocampus decreased at 6 and 9 months after radiotherapy. FA in the ipsilateral hippocampus before radiochemotherapy decreased compared with 6 months after radiotherapy. Compared to the end of radiotherapy, as well as 3- and 6-months post-radiotherapy, the regional cerebral blood volume (rCBV) in the genu of the corpus was significantly lower at 12 months post-radiotherapy. Some MRI parameters in different regions of the brain were negatively correlated with the mean and maximum dose. There was no significant effect of radiochemotherapy on the neurocognitive functioning of patients with HGGs found before radiochemotherapy until 12 months after radiotherapy. The radiation-induced FA decrease in the bilateral hippocampus preceded cognitive dysfunction, and DTI of the hippocampus may provide a useful biomarker for predicting radiation-induced neurocognitive impairment in patients with HGGs.

## Introduction

High-grade gliomas (HGG) are solid malignant tumours that arise from the uncontrolled differentiation of astrocytes and may be classified as grade III anaplastic astrocytoma, oligodendroglioma, or oligoastrocytoma, along with grade IV glioblastoma multiforme. HGGs account for the majority of primary brain tumours of the central nervous system (CNS). The prognosis of patients with glioblastoma is poor with an average duration of survival that is less than 12 months^1,2^. Treatment of HGG aims to increase the overall survival time and quality of life of the patients. The standard of care for HGG patients is surgical resection of the primary tumour (if possible), followed by concurrent chemotherapy and radiation therapy. Currently, postoperative concurrent radiochemotherapy is often supplemented with adjuvant chemotherapy using temozolomide (TMZ) in patients with glioblastoma^3^. While some studies have assessed the neurocognitive impairments of patients with low-grade gliomas, there is minimal information about the neurocognitive functioning of patients with HGGs after radiochemotherapy in the current literature. On the one hand, patients with HGGs have shorter overall survival, especially for patients with glioblastomas as their median overall survival is less than 12 months. Since cognitive impairment following radiochemotherapy is a late stage radiation injury, some patients with HGG die without impairments in their cognitive function. However, HGGs are more likely to recur, and the recurrence has been shown to negatively impact the cognitive assessment and cognitive functioning of patients.

The neurocognitive functions of patients with HHGs who receive radiochemotherapy may be assessed using several techniques, such as the mini-mental state examination (MMSE)^4,5^. However, the sensitivity of the Montreal Cognitive assessment (MoCA) is higher than that of MMSE for evaluating mild cognitive impairment (MCI) in adults^6,7^. In addition, the MoCA was successfully employed to assess the cognitive functioning of patients with brain metastases after radiotherapy^8^. Event-related potentials (ERPs), which are small voltages generated in the brain in response to stimuli, have been used for the assessment of patients with dementia in the past. The P300 element of the ERPs refers to specific mental activities, such as the rapid recognition and classification of multidimensional stimuli, along with stimuli expectancy and recovery from the short-term memory. Many regions in the brain can generate the ERP wave, including the temporal and parietal lobes, along with the hippocampus^9^. ERPs have been used in studies of MCI, and have been thought to be correlated with MMSE^10^.

Diffusion tensor imaging (DTI) is a newer neuroimaging technology, based off the principles of MRI, that can be used to measure the diffusion and directionality of water molecules in biological tissues. This allows for the quantitative analysis of alterations in metabolite levels and water diffusivity parameters within visually normal-appearing white matter (NAWM) during conventional MRI^11^. Some parameters of DTI, along with the apparent diffusion coefficient (ADC) and fractional anisotropy (FA), may be used to assess NAWM changes in patients with gliomas who undergo radiotherapy^12,13^. Perfusion weighted imaging (PWI) is another MRI-based functional imaging modality that uses to an endogenous or exogenous contrast agent for identifying brain regions that contain sufficient blood flow for the maintenance of structural integrity. PWI is also used to assess the capillary microcirculation of tissues and tissue microvascular density through the measurement of relative cerebral blood volume (rCBV) and cerebral blood flow (CBF)^14,15^. PWI has been used to evaluate changes in perfusion and amnestic MCI in the posterior cingulate region when combined with magnetic resonance spectroscopy (MRS), DTI, or F-18 fluorodeoxyglucose (^18^F-FDG) positron emission tomography (PET) imaging in patients with Alzheimer’s disease, and the parameters mentioned above were found to be correlated with the neuropsychological testing results^16,17^. However, two studies have reported that radiotherapy can adversely impact the cognitive functions of patients with HGG^18,19^. Additional studies are needed to determine the biological mechanisms by which radiation can cause neurocognitive impairments in patients with HGG.

In this study, HGG patients undergoing postoperative radiochemotherapy were recruited to measure changes in cognitive function using MMSE, MoCA, and ERP-P300 before and after radiochemotherapy at different time points. DTI and PWI were performed to evaluate changes in DTI and PWI parameters in different regions of the brain at different intervals. Finally, correlations between the parameters of functional MRI and radiation dose in the same regions of the brain were analysed to search for radiographic indexes to predict the possibility of MCI in patients with HGG who undergo postoperative radiochemotherapy, which will allow for the optimization of radiotherapy treatment plans.

## Methods

### Patient selection

The protocol for this study was reviewed and approved by the Medical Ethics Committee of Xiangya Hospital (Central South University, Changsha, Hunan, P.R. China). The research design and methods described in this study were performed in accordance with the regulations and procedures for human patient protection laws, including the Good Clinical Practice (GCP) and International Conference on Harmonization of Technical Requirements for Registration of Pharmaceuticals for Human USE-GCP (ICH-GCP). All of the subjects were required to provide written informed consent before being included in the study. In total, 23 patients with pathologically-confirmed HGG, as classified in the World Health Organization 2016 Classification of Tumours of the CNS^20^, were enrolled in this prospective study at Xiangya Hospital of Central South University from May 2014 to November 2014. All patients were screened for the isocitrate dehydrogenase-1 (IDH-1) by immunohistochemistry (IHC) for IDH1-R132H and DNA sequencing for IHC-negative patients by the Department of Pathology at Xiangya Hospital of Central South University. All cases were reviewed by three senior neuropathologists. For IHC-negative cases, IDH mutations were based on DNA sequencing.

All patients underwent craniotomy and radiochemotherapy and had not undergone previous radiation or chemotherapy treatment. The HGG patients underwent simultaneous integrated boost (SIB) intensity-modulated radiotherapy (IMRT) before therapy^21^. The patients recruited in this study were not treated with postoperative antidepressant drugs. Four patients were removed from the study due to radiographically-identified disease recurrence, while one other patient was removed from the study at 3 months post-radiotherapy at the patient’s request. The average age was 44.22 ± 11.92 years (range 18 to 65 years). The data from 18 patients were assessed, which included 10 males and 8 females with an average education level of 9.7 ± 3.6 years of education (range 6–16 years). Lesions were located above the tentorium of the cerebellum, and all subjects were dextral. Excluded from this study were patients with apparent diminished capacity, family history of psychiatric or neurological illness, severe hypertension, heart disease, and severe dyslipidemia. Clinical data of the final patients enrolled in the study are shown in Table 1.

**Table 1:** Clinical characteristics of 18 patients with high-grade gliomas.

Number	Gender	Age (years)	Histological classification	IDH	Location	Duration of education years	Extent of resection	Seizures	Antiepileptic drug usage
1	Male	23	III	Wildtype	L lateral ventricles	9	TR	Yes	Sodium Valproate
2	Male	44	III	Mutant	R frontal lobe	9	STR	None	Sodium Valproate
3	Male	41	III	Wildtype	R frontal lobe	9	PR	None	None
4	Male	35	IV	Wildtype	L thalamus	16	PR	Yes	Levetiracetam
5	Female	60	III	Mutant	R parieto-fronto-temporal lobe	6	TR	None	None
6	Female	33	IV	Wildtype	L frontal lobe	12	STR	Yes	Sodium Valproate
7	Male	45	IV	Wildtype	R frontal lobe	6	TR	Yes	None
8	Female	47	III	Mutant	R frontal lobe	6	STR	Yes	None
9	Female	51	III	Mutant	L parietal lobe	6	STR	None	None
10	Male	56	IV	Wildtype	R frontal lobe	12	TR	None	None
11	Male	36	IV	Wildtype	L frontal lobe	12	PR	None	Sodium Valproate
12	Female	26	IV	Wildtype	L temporal lobe	16	STR	Yes	None
13	Female	40	III	Mutant	R fronto-temporal-insular lobe	9	PR	None	None
14	Male	31	III	Mutant	L parieto-temporal lobe	9	STR	Yes	Sodium Valproate
15	Male	58	IV	Wildtype	R lateral ventricles	6	STR	None	None
16	Female	25	III	Mutant	L frontal lobe	16	TR	Yes	Sodium Valproate
17	Male	57	IV	Wildtype	R frontal lobe	6	TR	None	None
18	Female	52	IV	Wildtype	L frontal lobe	9	STR	None	None

R: right, L: left, TR: total resection, STR: subtotal resection, PR: partial resection, WHO: world health organization, IDH: isocitrate dehydrogenase.

### Radiotherapy and chemotherapy

All patients received postoperative IMRT with concurrent or adjuvant chemotherapy of TMZ. The targets for radiation therapy were created according to the guidelines provided by the European Organization for Research and Treatment of Cancer (EORTC)^22^. Gross tumour volume (GTV) was delineated using contrast-enhanced T1 MR images. The clinical target volume 1 (CTV1) was deemed as the GTV with a 1.5 cm margin, while the 2.5 cm margin was the clinical target volume 2 (CTV2). CTV1 and CTV2 with 0.3–0.5 cm margins were defined as planning target volume 1 (PTV1) and PTV2. While PTV1 consisted of 60 Gy in 30 fractions (2 Gy per fraction), PTV2 consisted of 54 Gy in 30 fractions (1.8 Gy per fraction) using the simultaneous IBT. The means of the maximum dose (D_max_) and dose (D_mean_), which were applied to the different brain regions, are described in Table 2. Each patient in this study received concurrent chemotherapy, which consisted of TMZ (75 mg/m^2^/day), along with IMRT and adjuvant chemotherapy with up to six cycles of maintenance TMZ (150–200 mg/m^2^/day on days 1–5 repeated every 28 days).

**Table 2:** Comparison of MMSE, MoCA and ERP-P300 at different time points.

		Pre-RCT	End of RT	3 mons post- RT	6 mons post- RT	9 mons post-RT	12 mons post-RT	F	*p*
Cognitive function(Mean ± SD)	MMSE	27.61 ± 2.75	28.11 ± 2.47	28.17 ± 2.57	27.22 ± 3.04	27.28 ± 2.5	26.56 ± 3.93	0.79	0.56
	MoCA	23.00 ± 5.37	24.94 ± 4.98	23.56 ± 5.90	22.83 ± 5.32	22.50 ± 6.92	23.11 ± 5.65	0.41	0.84
ERP- P300 (Mean ± SD)	Amplitude (μV)	11.21 ± 6.61	14.64 ± 8.72	13.46 ± 7.15	13.89 ± 6.03	13.58 ± 2.92	13.76 ± 4.67	0.63	0.68
	Latency (mS)	345.78 ± 36.25	353.44 ± 45.14	352.28 ± 39.09	343.39 ± 27.91	344.11 ± 30.29	347.00 ± 31.32	0.26	0.94

MMSE: Mini mental status examination, MoCA: Montreal cognitive assessment, RCT: radiochemotherapy, RT: radiotherapy, mS: millisecond, μV: microvolt, ERP-P300: event-related potential P300, pre-RCT: before radiochemotherapy, post-RT: after radiotherapy, mons: months, SD: standard deviation.

### Cognitive function tests

The cognitive functions of 18 patients with HGG treated with postoperative radiochemotherapy were evaluated before and at the end of IMRT, and 3, 6, 9, and 12 months after IMRT using MMSE^23^, MoCA^8^, and ERP-P300^10^. Cognitive functions were assessed before the MRI examinations were performed.

### DTI and PWI

The clinical HDx 3.0 T General Electric (GE) Signa scanner (GE Medical Systems, Milwaukee, WI, USA) with an 8-channel head coil was used to acquire the MRI data. The routine brain protocol for MRI included axial T1-weighted images (repetition time [TR]/echo time [TE], 600/15 ms) and T2-weighted images (TR/TE, 5200/140 ms) were obtained for each patient for the detection of recurrent intracranial lesions. DTI was performed with a single-shot echo planar imaging (EPI) spin-echo sequence (TR = 2046 ms, TE = 87 ms, field of view [FOV] = 25.6 by 25.6 mm^2^, matrix = 128 by 128, slice thickness = 3 mm, gap = 2 mm). The diffusion sensitising gradient was applied in 25 directions with a diffusion-weighted factor b-value of 1000 s/mm^2^. Next, 50 images were acquired in 32 non-collinear directions using a scanner time of 7 min and 12 s.

During the next step, 50 repetitions of perfusion-weighted EPI sequences (TR = 1600 ms; TE = minimum; FOV = 24 by 24 mm^2^; matrix = 128 by 128; 19 axial slices; slice thickness = 5 mm; gap = 1.5 mm) were obtained after the patient received 20 mL of gadolinium diethylenetriamine penta-acetic acid (gadolinium-DTPA) injected at a rate of 4 mL/s. The conditions included: contrast agent, dimeglumine gadopentetate; dose, 0.2 mL/kg; 90° flip angle; scanner time, 1 min 20 s; injection time: 1 min 10 s. Dynamic susceptibility contrast techniques were used.

The imaging data were processed with the Ancillary Advantage Workstation Version 4.2 (ADW4.2) from GE Medical Systems (Milwaukee, WI, USA). The MR DTI files were transferred to the Advantage Workstation (GE Healthcare, Chicago, IL, USA) for data processing. FA measurements in the white matter were conducted for multiple regions of the brain, including the contralateral frontal and temporal lobes, posterior cingulate gyrus, genu of the corpus callosum, splenium of the corpus callosum, and grey matter of the bilateral hippocampus. Next, DTI and PWI were conducted before and after the completion of radiochemotherapy, and subsequently every 3 months through 12 months. Three regions of interests (ROIs) with an approximately equal area of 20–40 mm^2^ were created in different brain regions of each patient. Precautions were taken to avoid placing the ROIs in areas of tissue necrosis or cystic degeneration. The ROIs were independently evaluated by two experienced neuro-radiologists to assess their eligibility and disagreement were resolved by a consensus. The FA average of the three ROI values above the brain regions was accepted as the final value. The average of regional cerebral blood flow (rCBF), regional cerebral blood volume (rCBV), time to peak (TTP), and mean transit time (MTT) were measured for the brain regions mentioned above using a similar approach.

### Statistical analysis

The Statistical Package for Social Science (SPSS) Version 17.0 (IBM, Chicago, IL, USA) was used for the statistical analyses. The parameters of the cognitive function and functional MRI were expressed as the mean ± standard deviation. All variances were analysed by Mauchly’s test of sphericity (*p* > 0.05). The one-way analysis of variance (ANOVA) was performed to investigate the homogeneity of variance, along with the least-significant difference test to find statistically significant differences in MMSE, MoCA, amplitude and latency of ERP-P300, FA, rCBF, rCBV, MTT, and TTP in specific brain regions at different time points. Pearson’s correlation coefficient was employed to show the relationship between D_max_ and D_mean_ and FA, rCBF, rCBV, rMTT, and TTP in specific brain regions at different time points. *P*-values that were < 0.05 were considered statistically significant.

## Results

### Comparison of MMSE, MoCA, and ERP-P300 at different time points

**Figure 1:**
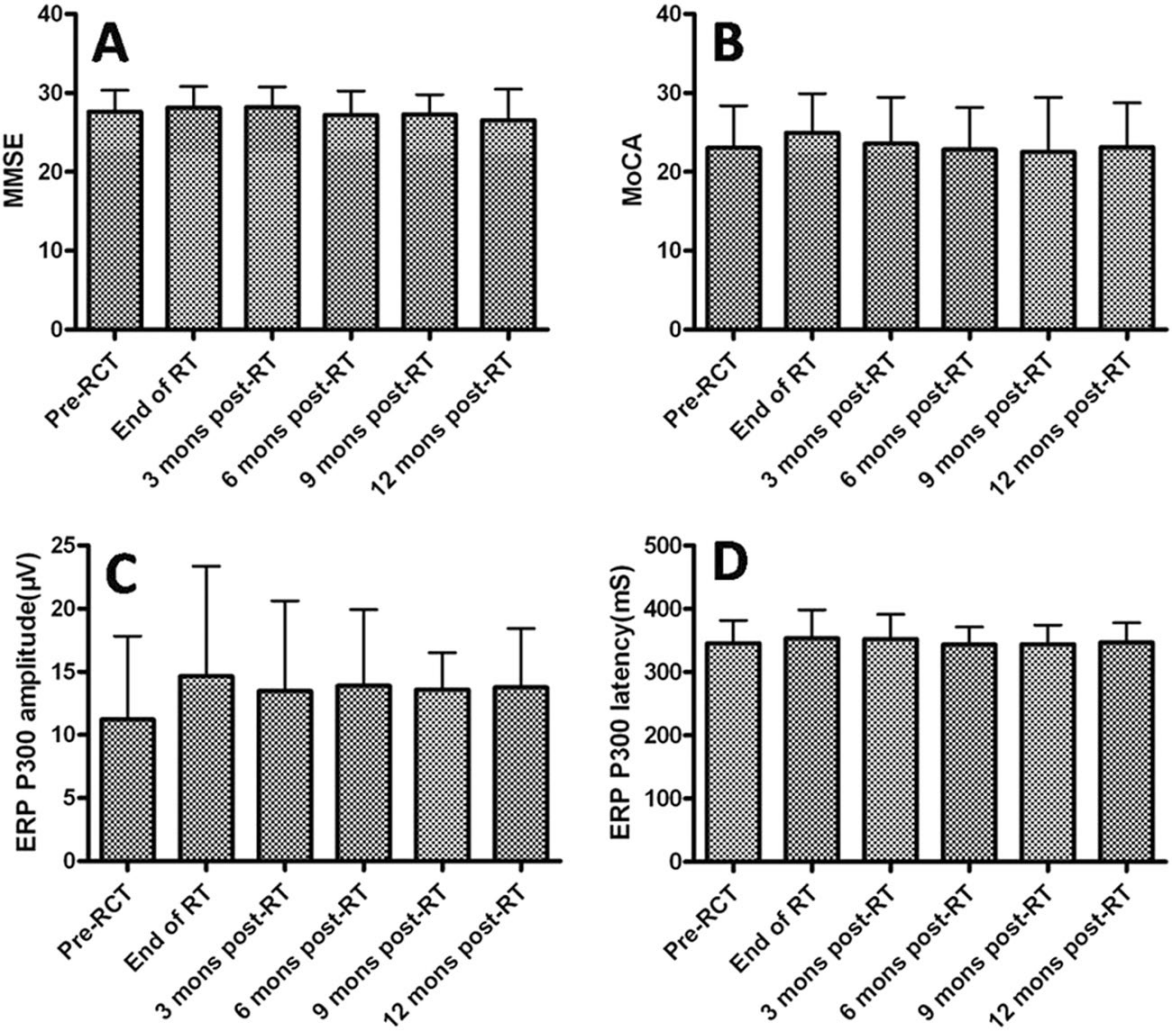
MMSE: Mini mental status examination, MoCA: Montreal Cognitive Assessment, Mons: Months, RCT: Radiochemotherapy, RT: Radiotherapy, mS: millisecond, μV: microvolt, ERP- P300: Event-related potential P300.

The differences in MMSE before and after IMRT, or at 3, 6, 9, and 12 months after IMRT were insignificant for the patients with HGGs (F = 0.79, *p* = 0.56). There were no significant differences in MoCA before and after IMRT, or at 3, 6, 9, and 12 months after IMRT (F = 0.41, *p* = 0.84). In addition, there were no statistically significant differences in the amplitude or latency of ERP-P300 levels before IMRT, after IMRT, or at 3, 6, 9, and 12 months after IMRT (F = 0.63, *p* = 0.68 and F = 0.26, *p* = 0.94, respectively), as shown in Fig. 1 and Table 2.

### Comparison of FA, rCBV, rCBF, MTT, and TTP at different time points

**Figure 2:**
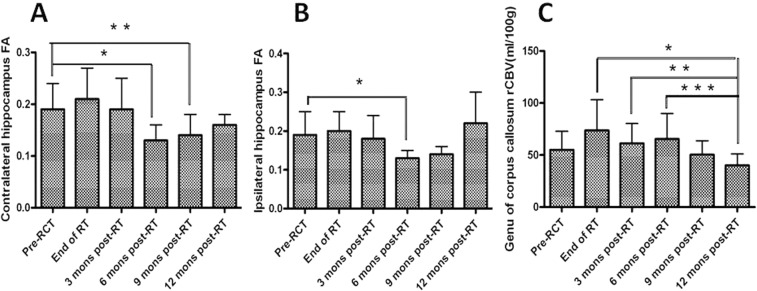
FA: fractional anisotropy, rCBV: regional cerebral blood volume, RCT: radiochemotherapy, RT: radiotherapy, mons: months, (**A**) FA of contralateral hippocampus at different time points, *FA pre-RCT vs FA 6 mons post-RT, *p* < 0.001, **FA pre-RCT vs FA 9 mons post-RT, *p* = 0.009. (**B**) FA of ipsilateral hippocampus at different time points, *FA pre-RCT vs FA 9 mons post-RT, *P* = 0.005. (**C**) rCBV of genu of corpus callosum at different time points, *rCBV at end of RT vs rCBV 12 mons post-RT, *p* = 0.003, **rCBV 3 mons post-RT vs rCBV 12 mons post-RT, *P* = 0.044, ***rCBV 6 mons post-RT vs rCBV 12mons post-RT, *p* = 0.021.

Significant differences in the FA of the contralateral and ipsilateral hippocampi were detected between the groups at different time points (F = 7.321, *p* < 0.001; and F = 2.866, *p* = 0.021, respectively). The rCBV in the genu of the corpus callosum was also significantly different between groups at different time points (F = 2.547, *p* = 0.037). FA in the contralateral hippocampus 6 and 9 months after radiotherapy decreased significantly compared with that before radiochemotherapy from 0.19 ± 0.05 to 0.13 ± 0.003 and 0.14 ± 0.004, respectively (*p* < 0.001 and *p* = 0.009, respectively). FA in the ipsilateral hippocampus before radiochemotherapy decreased significantly compared with that 6 months post-radiochemotherapy from 0.19 ± 0.06 to 0.13 ± 0.02 (*p* = 0.005). Until 1 year after radiotherapy, FA in the bilateral hippocampus was restored and was not significantly different from that before radiochemotherapy. At 1 year post-radiotherapy, the rCBV in the genu of the corpus callosum was significantly lower than the rCBV detected at the end of radiotherapy, as well as 3 and 6 months post-radiotherapy (40.08 ± 10.92 vs. 73.66 ± 29.57, *p* = 0.003; 40.08 ± 10.92 vs. 61.35 ± 18.92, *p* = 0.044; and 40.08 ± 10.92 vs. 65.42 ± 24.68, *p* = 0.021, respectively). A downward trend in the rCBV was detected in the genu of the corpus callosum between before radiotherapy and 12 months post-radiotherapy. However, this downward trend was not statistically significant (55.05 ± 40.08 vs. 40.08 ± 10.92, *p* = 0.153; Fig. 2 and Table 3).

**Table 3:** Comparison of the parameters of DTI and PWI in different time points at different brain regions.

Regions of brain	Parameters of MRI(Mean ± SD)	Pre-RCT	End of RT	3 mons post-RT	6 mons post-RT	9 mons post-RT	12 mons post-RT	F	*p*
Contralateral frontal lobe	FA	0.28 ± 0.09	0.29 ± 0.07	0.31 ± 0.09	0.35 ± 0.09	0.41 ± 0.07	0.30 ± 0.09	2.282	0.058
	rCBF(ml/100 g/min)	681.35 ± 339.34	668.69 ± 321.18	625.14 ± 325.12	605.34 ± 208.25	791.18 ± 236.86	726.02 ± 239.84	0.391	0.853
	rCBV(ml/100 g)	41.43 ± 13.41	51.49 ± 18.98	44.30 ± 22.39	47.02 ± 20.51	37.69 ± 10.99	37.65 ± 19.00	0.761	0.582
	MTT(s)	4.96 ± 2.65	5.75 ± 3.35	5.34 ± 3.28	5.26 ± 2.28	2.99 ± 0.94	2.83 ± 0.85	1.550	0.188
	TTP(s)	21.55 ± 7.95	23.23 ± 7.56	23.06 ± 7.27	21.65 ± 5.95	18.43 ± 7.21	15.80 ± 4.81	1.256	0.295
Contralateral temporal lobe	FA	0.32 ± 0.08	0.35 ± 0.09	0.32 ± 0.12	0.37 ± 0.11	0.33 ± 0.07	0.37 ± 0.09	0.646	0.666
	rCBF(ml/100 g/min)	732.59 ± 420.55	635.44 ± 308.56	567.53 ± 289.73	652.19 ± 244.11	698.92 ± 112.85	751.08 ± 278.70	0.555	0.734
	rCBV(ml/100 g)	46.86 ± 19.73	56.39 ± 21.93	50.99 ± 37.43	46.78 ± 28.82	40.34 ± 3.70	41.25 ± 18.44	0.451	0.811
	MTT(s)	4.47 ± 2.39	6.39 ± 3.98	6.01 ± 3.98	5.20 ± 3.22	3.41 ± 0.82	3.33 ± 0.86	1.412	0.233
	TTP(s)	21.54 ± 7.98	22.14 ± 8.45	21.60 ± 9.04	19.99 ± 6.31	24.12 ± 2.76	16.77 ± 3.81	0.675	0.644
Contralateral hippocampus	FA	0.19 ± 0.05	0.21 ± 0.06	0.19 ± 0.06	0.13 ± 0.03	0.14 ± 0.04	0.16 ± 0.02	7.231	<0.001
	rCBF(ml/100 g/min)	540.22 ± 149.98	639.04 ± 357.08	660.21 ± 446.17	462.06 ± 202.20	348.30 ± 238.88	652.70 ± 364.30	1.249	0.298
	rCBV(ml/100 g)	36.33 ± 17.61	68.52 ± 60.10	69.14 ± 80.63	44.87 ± 38.52	20.58 ± 7.30	32.03 ± 8.52	1.475	0.212
	MTT(s)	4.41 ± 2.17	6.81 ± 4.42	5.61 ± 3.60	5.55 ± 4.16	3.43 ± 0.89	3.48 ± 0.73	1.399	0.238
	TTP(s)	21.07 ± 5.84	24.66 ± 6.26	24.46 ± 7.00	21.48 ± 6.31	19.72 ± 4.55	19.86 ± 2.60	1.295	0.278
Ipsilateral hippocampus	FA	0.19 ± 0.06	0.20 ± 0.05	0.18 ± 0.06	0.13 ± 0.02	0.14 ± 0.02	0.22 ± 0.08	2.886	0.021
	rCBF(ml/100 g/min)	540.27 ± 195.15	690.19 ± 299.54	729.70 ± 475.67	691.10 ± 415.80	521.20 ± 186.60	618.73 ± 180.28	0.690	0.633
	rCBV(ml/100 g)	35.14 ± 19.14	63.00 ± 54.71	80.96 ± 79.67	47.68 ± 29.47	24.62 ± 8.93	36.77 ± 11.24	2.030	0.087
	MTT(s)	3.98 ± 1.82	5.58 ± 3.55	6.10 ± 3.27	4.84 ± 2.27	2.85 ± 0.64	3.78 ± 1.45	1.984	0.094
	TTP(s)	21.82 ± 5.92	23.16 ± 7.97	23.32 ± 9.52	23.80 ± 5.20	21.54 ± 4.38	18.01 ± 4.21	0.677	0.643
Posterior cingulate gyrus	FA	0.24 ± 0.06	0.24 ± 0.07	0.26 ± 0.07	0.26 ± 0.05	0.30 ± 0.03	0.30 ± 0.04	1.888	0.110
	rCBF(ml/100 g/min)	717.78 ± 211.76	714.38 ± 256.81	742.38 ± 302.87	701.59 ± 214.94	924.72 ± 192.35	950.77 ± 291.34	1.431	0.227
	rCBV(ml/100 g)	68.68 ± 91.80	70.82 ± 82.75	73.27 ± 76.08	70.73 ± 84.47	48.54 ± 15.28	72.82 ± 30.08	0.083	0.995
	MTT(s)	3.70 ± 1.60	4.50 ± 2.18	4.58 ± 1.86	4.03 ± 0.64	2.51 ± 0.56	2.91 ± 0.39	2.261	0.060
	TTP(s)	19.39 ± 5.55	19.71 ± 5.57	17.85 ± 5.53	18.45 ± 3.96	13.96 ± 6.18	15.45 ± 6.86	1.250	0.298
Genu of corpus callosum	FA	0.43 ± 0.21	0.47 ± 0.24	0.47 ± 0.20	0.5 ± 0.18	0.45 ± 0.11	0.43 ± 0.18	0.270	0.928
	rCBF(ml/100 g/min)	1069.74 ± 487.88	1303.23 ± 400.11	1222.92 ± 504.29	1293.51 ± 441.57	1011.32 ± 225.75	1035.00 ± 423.12	0.808	0.548
	rCBV(ml/100 g)	55.05 ± 17.70	73.66 ± 29.57	61.35 ± 18.92	65.42 ± 24.68	50.34 ± 13.36	40.08 ± 10.92	2.547	0.037
	MTT(s)	3.58 ± 1.96	3.64 ± 2.10	3.30 ± 1.84	3.68 ± 2.41	3.02 ± 0.74	2.48 ± 0.53	0.446	0.810
	TTP(s)	18.20 ± 6.97	17.38 ± 9.11	17.09 ± 7.61	13.71 ± 6.06	14.91 ± 6.41	14.48 ± 8.24	0.667	0.650
Splenium of corpus callosum	FA	0.63 ± 0.15	0.67 ± 0.15	0.63 ± 0.20	0.67 ± 0.18	0.75 ± 0.06	0.74 ± 0.09	0.713	0.616
	rCBF(ml/100 g/min)	1485.54 ± 444.86	1811.63 ± 375.19	1490.15 ± 547.90	1557.48 ± 767.90	1645.20 ± 196.64	1368.22 ± 608.16	0.814	0.545
	rCBV(ml/100 g)	86.69 ± 50.02	103.06 ± 118.47	61.21 ± 19.94	71.15 ± 33.95	57.47 ± 8.97	88.72 ± 50.11	0.867	0.509
	MTT(s)	3.23 ± 1.78	2.93 ± 1.97	2.92 ± 1.48	2.81 ± 1.73	2.08 ± 0.27	3.48 ± 1.90	0.480	0.790
	TTP(s)	13.85 ± 7.82	13.25 ± 10.03	13.26 ± 8.73	14.48 ± 7.91	10.36 ± 4.12	11.33 ± 4.77	0.264	0.931

MRI: magnetic resonance imaging, mons: months, RCT: radiochemotherapy, RT: radiotherapy, pre-RCT: before radiochemotherapy, post-RT after radiotherapy, FA: fractional anisotropy, rCBF: regional cerebral blood flow, rCBV: regional cerebral blood volume, MTT: mean transit time, TTP: time to peak, s: second, SD: standard deviation.

### Correlation analysis between DTI parameters and D_max_ and D_mean_ of different regions of the brain

The D_max_ and D_mean_ of the contralateral frontal and temporal lobes, genu of the corpus callosum, bilateral hippocampus, splenium of the corpus callosum, and posterior cingulate gyrus were contoured and assessed using the Eclipse Planning System (Varian, Palo Alto, CA, USA). Mean D_max_ and D_mean_ values were calculated and are shown in Table 4. Pearson’s correlation analysis was performed with the D_max_ and D_mean_ of the organs mentioned above and corresponding FA.

**Table 4:** Mean D_max_ and mean D_mean_ of brain regions.

	Contralateral frontal lobe	Contralateral temporal lobe	Contralateral hippocampus	Ispilateral hippocampus	Posterioral cingulate gyrus	Genu of corpus callosum	Splenium of corpus callosum
Mean D max(Gy, Mean ± SD)	55.62 ± 14.64	46.94 ± 11.48	41.49 ± 12.85	51.22 ± 16.02	54.98 ± 16.66	55.82 ± 15.46	47.54 ± 12.59
Mean Dmean (Gy, Mean ± SD)	36.97 ± 16.57	24.81 ± 10.69	31.26 ± 11.90	42.90 ± 16.94	50.13 ± 19.08	52.23 ± 16.83	40.27 ± 13.67

D_max_: maximum dose, D_mean_: mean dose, SD: standard deviation.

By the end of radiotherapy, there was a negative correlation between the FA in the contralateral temporal lobe and D_max_ in the contralateral temporal lobe (r = −0.571, *p* = 0.028). There was also a negative correlation between the FA in the splenium of the corpus callosum and D_mean_ in the splenium of the corpus callosum (r = −0.489, *p* = 0.039). There was no correlation in FA values between the radiation dose and the responding brain regions, including the posterior cingulate gyrus, genu of the corpus callosum, contralateral frontal lobe, and bilateral hippocampus (all *p* > 0.05; Supplementary Table S1).

At 3 months post-radiotherapy, the FA in the ipsilateral hippocampus was found to be negatively correlated with the D_max_ and D_mean_ in the ipsilateral hippocampus (r = −0.683, *p* = 0.002 and r = −0.532, *p* = 0.023, respectively). In addition, the FA in the genu of the corpus callosum was found to be negatively correlated with the D_mean_ in the same region of the brain (r = −0.499, *p* = 0.035). FA values in the contralateral frontal and temporal lobes, contralateral hippocampus, posterior cingulate gyrus, and splenium of the corpus callosum were not correlated with radiation dose in the responding brain regions (all *p* > 0.05; Supplementary Table S2).

Six months after radiotherapy, the D_max_ in the ipsilateral hippocampus was found to negatively correlate with the FA in the ipsilateral hippocampus (r = −0.477, *p* = 0.045). FA of the posterior cingulate gyrus was negatively correlated with D_max_ and D_mean_ in the posterior cingulate gyrus (r = −0.576, *p* = 0.012, and r = −0.608, *p* = 0.007, respectively). FA in the contralateral frontal and temporal lobes, contralateral hippocampus, genu of the corpus callosum, and splenium of the corpus callosum was not correlated with radiation dose in the responding brain regions (*p* > 0.05; Supplementary Table S3).

Nine months after radiotherapy, FA values in all regions of the brain studied were not correlated with the radiation dose in the responding brain regions (all *p* > 0.05; Supplementary Table S4).

At 12 months post-radiotherapy, the D_max_ and D_mean_ in the contralateral frontal lobe showed a negative correlation with the FA in the contralateral frontal lobe (r = −0.590, *p* = 0.010, and r = −0.504, *p* = 0.033, respectively). FA in the posterior cingulate gyrus was negatively correlated with D_max_ and D_mean_ in the posterior cingulate gyrus (r = −0.816, *p* < 0.001, and r = −0.800, *p* < 0.001, respectively). The radiation dose in the responding brain regions was not correlated with the FA in the splenium of the corpus callosum, bilateral hippocampus, contralateral temporal lobe, or genu of the corpus callosum (all *p* > 0.05; Supplementary Table S5).

### Correlation analysis between PWI parameters and D_max_ and D_mean_ of the different brain regions

The rCBV in the contralateral frontal lobe was positively correlated with D_max_ in the contralateral frontal lobe at the end of radiotherapy (r = 0.986, *p* = 0.006). This parameter was not correlated with the radiation dose in the responding brain regions at 3, 6, 9, and 12 months following radiotherapy (all *p* > 0.05; Supplementary Tables S1–S5).

The rCBF showed a negative correlation with both D_max_ and D_mean_ in the genu of the corpus callosum. The data at 6 months after radiotherapy (r = −0.500, *p* = 0.034; and r = −0.524, *p* = 0.026, respectively), 9 months after radiotherapy (r = −0.653, *p* = 0.003; and r = −0.681, *p* = 0.002, respectively), and 12 months after radiotherapy (r = −0.726, *p* = 0.001; and r = −0.747, *p* < 0.001, respectively) show statistical significance (Supplementary Tables S3–S5).

MTT had implications exclusively at 3 months after radiotherapy. MTT in the posterior cingulate gyrus was found to be negatively associated with D_max_ and D_mean_ in the same region (r = −0.547, *p* = 0.019; and r = −0.481, *p* = 0.044, respectively). Similarly, MTT in the genu of the corpus callosum was found to be negatively associated with D_max_ and D_mean_ in the same region (r = −0.597, *p* = 0.009; and r = −0.552, *p* = 0.018, respectively), as shown in Supplementary Table S2.

Three months after radiotherapy, TTP of the posterior cingulate gyrus was negatively correlated with D_max_ and D_mean_ in the posterior cingulate gyrus (r = −0.583, *p* = 0.011; and r = −0.513, *p* = 0.030, respectively). TTP in the splenium of the corpus callosum was negatively correlated with D_mean_ in the splenium of the corpus callosum (r = −0.559, *p* = 0.016). Six months after radiotherapy, TTP in the contralateral temporal lobe was found to be negatively correlated with the D_max_ in the same region of the brain (r = −0.576, *p* = 0.012). TPP in other areas of the brain did not correlate with the radiation dose in the responding brain regions at any other point of time (all *p* > 0.05; Supplementary Tables S2, S3).

## Discussion

In this retrospective study, we investigated changes in the cognitive function of patients with HGGs undergoing radiochemotherapy for up to 12 months after completing radiotherapy. No significant differences in the MMSE, MoCA, or ERP-P300 were revealed in patients with HGG before radiochemotherapy, after radiotherapy, or at 3, 6, 9, and 12 months post-radiotherapy. Previously, Taylor *et al*. stated that there was no clear evidence of cognitive impairment in glioma patients who had undergone radiochemotherapy. This study also assessed the cognitive function of non-progressive patients using MMSE. This study is superior in terms of longer study time (24 months) and larger sample size (550 of 701 HGG patients). The same conclusions demonstrated that cognitive worsening did not emerge at the level of MMSE^4^. However, MMSE may lack the sensitivity require to detect MCI. For example, Meyers *et al*. found that MMSE lacked the sensitivity to detect frontal-subcortical network dysfunction, which is often associated with radiation therapy in the brain, and suggested that studies using this screening technique may be unable to detect mild cognitive changes^24^. In another study, Brown *et al*. assessed the cognitive function of patients with low-grade glioma who had an average follow-up of 7.4 years. A limited number of patients in the study showed signs of impaired cognitive function after undergoing radiotherapy. However, the decline in cognitive function was not apparent with the MMSE, so the researchers suggested that a more thorough cognitive assessment might have revealed a more substantial reduction in cognitive function^25^. Keime-Guibert *et al*. assessed the cognitive function of elderly patients who were divided into two treatment groups, including supportive therapy-alone or supportive therapy in combination with radiotherapy, with the median follow-up period of 21 weeks. The MMSE scores were of both groups were significantly lower than those of the control group (*p* = 0.007), yet there was no significant difference between the two treatment groups (*p* = 0.13), indicating that the radiotherapy-alone did not cause the decline of cognitive function^26^.

In this study, we concluded that radiochemotherapy did not obviously affect the cognitive status of HGGs at least within the first year. However, there have been reports of moderate-to-severe cognition impairments in long-term HGG survivors. For example, Archibald *et al*. demonstrated the presence of cognition dysfunction in patients early after undergoing radiotherapy for HGG, and most patients are unable to living independently^18^. In another study. Schmidinger *et al*. assessed several factors, such as overall quality of life, psychophysiological variables, and cognitive dysfunction, in patients still alive 18 months after being diagnosed with gliomas. From the 18 patients in the study, 11 showed high satisfaction with their quality of life despite impairments in their psychophysiological and cognitive abilities^19^. The reasons cause the different cognitive functions evaluated in HGG after radiochemotherapy, which were related to different methods or the panel used to evaluate the cognitive status, different chemotherapy regimen, radiation dose and fractions, the effect of different extent of surgery and tumour recurrence and infiltration on cognitive function. Furthermore, the different intervals between the end of radiochemotherapy to the assessment of cognitive function affects the cognitive status. Our results indicated MMSE and MoCA at the end of radiotherapy and 3 months after radiotherapy were slightly higher than those before radiochemotherapy, although this difference was not statistically significant and may be related to the recovery of cognitive deficits after surgery and shrinkage of residual tumours following radiochemotherapy.

Our study indicated FA in the contralateral hippocampus 6 and 9 months after radiotherapy was significantly decreased compared with that before radiochemotherapy (*p* < 0.001 and *p* = 0.009, respectively). The FA in the ipsilateral hippocampus before radiochemotherapy was significantly lower when compared with that at 6 months post-radiotherapy from 0.19 ± 0.06 to 0.13 ± 0.02 (*p* = 0.005), and 6 months after radiotherapy, and the FA in the ipsilateral hippocampus was negatively correlated with D_max_ in the ipsilateral hippocampus (r = −0.477, *p* = 0.045). Until 12 months after radiotherapy, the FA in the bilateral hippocampi was restored and was not significantly different from those before radiochemotherapy.

It has been suggested that the cognitive impairments caused by radiation exposure may result from radiation-induced injuries to the proliferating neuronal progenitor cells found in the subgranular zone of the hippocampus^27,28^. The hippocampus is vulnerable to damage from irradiation^29^. While small doses of radiation exposure can induce the apoptosis of neurons in the subgranular zone of small rodents^27^, the cerebrum remains largely unaffected by radiation exposure^29^. Radiation exposure has also been shown to induce the long-term reduction of neurogenesis in the subgranular zone^30^. Previously, Pospisil *et al*. investigated a potential connection between changes in memory and hippocampal N-acetylaspartate (h-tNAA) levels in metastatic brain cancer patients who received whole brain radiotherapy (WBRT)^31^. The h-tNAA is a neuronal density and cell viability marker that can be measured by ^1^H-MR spectroscopy. The findings showed correlations between left h-tNAA levels and auditory verbal learning test (AVLT) recognition and the revised subtests of the brief visuospatial memory test, along with another relationship between the right h-tNAA and AVLT total-recall.

Previously. Kumar *et al*. demonstrated how whole-body radiation could affect memory function, which is dependent on the hippocampus, and various microstructural alterations in the entire brain. To demonstrate these changes, 6-month old female C57BL/6 mice were exposed to whole-body gamma radiation at 2, 5, or 8 Gy. Visualization of parenchymal damage was accomplished with DTI at 48 h post-irradiation. Mice in the 5 Gy (*p* < 0.05) and 8 Gy (*p* < 0.001) groups showed hippocampal FA values that were significantly lower than those of the control group^32^. This research showed that the hippocampus is vulnerable to radiation damage, and even small doses of radiation cause damage to hippocampal regions of young mice. Moreover, FA is a sensitive parameter in response to hippocampal damage.

Dellani *et al*. performed prospective MR examinations, including DTI, in adult survivors of childhood acute lymphoblastic leukaemia who underwent chemoradiation 16 to 28 years prior to the study. The DTI findings revealed FA values in the hippocampi that were significantly lower (difference of 0.033 units, *p* < 0.001) when compared with an age-matched control group^33^. The reductions in FA values resulted from chemoradiation-induced structural changes in the brain. While degenerative brain disease is characterised by the loss of neurons, expanded intercellular space, and increased diffusivity^34^, radiation exposure results in the early apoptosis of oligodendrocytes^35,36^, along with alterations in the vasculature and a reduction in the number of periventricular progenitor cells^37^. FA may be a more sensitive marker for assessing primary myelination damage than mean diffusivity. Other studies have also shown that FA decreases once radiation has been terminated, which further confirms that demyelination is an early adverse effect caused by radiation exposure^38^.

In a previous study, Bodensohn *et al*. reported on the strong correlations detected between the difference for “simple reaction tests” and the right hippocampal mean dose (*p* = 0.016), maximum dose (*p* = 0.033), and the equivalent uniform dose (*p* = 0.024). Additionally, the “figural memory test” was significantly correlated with the maximum radiation dose in the left hippocampus (*p* = 0.045) in HGG patients who received 3D conformal radiation therapy with concomitant or adjuvant chemotherapy using TMZ based on the methylation state of the O^6^-methylguanine-DNA methyltransferase promoter^39^. At this time, there have been limited studies about hippocampal sparing and most have been conducted in patients with brain metastases who underwent WBRT. In cases of hippocampal avoidance, improved functional outcomes with lower degrees of cognitive impairment have been obtained^40^.

Despite these findings, it remains uncertain whether hippocampal sparing can affect the neurocognitive functions of patients with HGG. Furthermore, stem cell niches can be found in the subgranular zone of the hippocampus, which could contribute to the high recurrence rates of gliomas^41,42^. Hence, the ipsilateral hippocampus can be included in the target volume, while the contralateral hippocampus can be disregarded^43^. It will be necessary to reveal in future prospective clinical trials whether patients with HGG can benefit from radiotherapy avoiding the hippocampus.

PWI can directly reflect the blood perfusion of tissue and indirectly reflect the distribution of microvessels. In a previous report, Luckhaus *et al*. showed that rCBF was decreased in the MCI when compared with normal subjects having natural cognitive abilities in the mesiotemporal (−23%), amygdala (−20%), and posterior cingulate (−15%). Interestingly, there was no further decline in cognitive function in patients with early Alzheimer’s disease^44^. Our data showed that rCBV in the genu of the corpus callosum 12 months after radiotherapy was significantly lower than at the end of radiochemotherapy, as well as 3 and 6 months after radiotherapy (40.08 ± 10.92 vs. 73.66 ± 29.57, *p* = 0.003; 40.08 ± 10.92 vs. 61.35 ± 18.92, *p* = 0.044; and 40.08 ± 10.92 vs. 65.42 ± 24.68, *p* = 0.021, respectively). At 12 months post- radiotherapy, the rCBF in the genu of the corpus callosum was found to be negatively correlated with the D_max_ and D_mean_ in the genu of the corpus callosum (r = −0.726, *p* = 0.001; and r = −0.747, *p* = 0.000, respectively). There was a downward trend in rCBV before radiochemotherapy and 12 months after radiotherapy, which was not statistically significant (55.05 ± 40.08 vs. 40.08 ± 10.92, *p* = 0.153). The results from this study suggest that the reduction of rCBV in the genu of the corpus callosum is likely associated with the dose of radiation delivered to the specific area of the brain. Previously, Farjam *et al*. used dynamic contrast-enhanced MRI in 27 patients with low-grade gliomas or benign tumours who were treated with 3D conformal radiotherapy or IMRT (median dose 54 Gy) from pre-radiochemotherapy to 18 months post-radiotherapy. There was a significant increase in the average transfer constant (K^trans^) from pre-radiotherapy to 1-month post-radiotherapy (*p* < 0.0004), which was significantly correlated with the sex (*p* < 0.0007) and age (*p* < 0.00004) of patients, with older females showing a more obvious response to the dose. Also, the vascular dose-response in the left hippocampus of females correlated significantly with memory function changes at both 6 months post-radiotherapy (r = −0.95, *p* < 0.0006) and 18 months post-radiotherapy (r = −0.88, *p* < 0.02). The conclusions from the study suggested that the early hippocampal vascular dose-response may be a biomarker of late neurocognitive dysfunction in adults^45^.

There are a few limitations of this study that should be addressed. The two primary limitations include the relatively small sample size of the study and the insufficient follow-up period of only 12 months for cognitive function and MRI. Secondly, this study did not include a typical control group. Additionally, intracranial HGGs were located in different regions and sides of the brain and the extent of surgical resection of the tumour differed, and thus radiotherapy to different regions of the brain did not have the same effect on cognitive function.

Our findings indicated that there was no significant reduction in cognitive function before and after radiotherapy at different time points analysed until 12 months in HGGs. After radiotherapy, FA in the bilateral hippocampus showed a temporary decline at 6 to 9 months after radiotherapy but recovered gradually to the level pre-radiochemotherapy by 12 months post-radiotherapy. Some DTI and PWI parameters in different regions of the brain were negatively correlated with the radiation dose. The radiation-induced decrease in FA in the bilateral hippocampus preceded cognitive dysfunction and supports the investigation of DTI of the hippocampus as a biomarker to predict radiation-induced neurocognitive impairment in HGGs.

## Supplementary information

Supplementary Information for Effect of radiochemotherapy on the cognitive function and diffusion tensor and perfusion weighted imaging for high-grade gliomas: A prospective study
