# Supplementary material for: Effect of radiochemotherapy on the cognitive function and diffusion tensor and perfusion weighted imaging for high-grade gliomas: A prospective study
Source: Sci Rep. 2019 Apr 12;9:5967. doi: 10.1038/s41598-019-42321-8 (PMC6461706; doi:10.1038/s41598-019-42321-8)
Supplement: Supplementary file 1 — Supplementary Information for Effect of radiochemotherapy on the cognitive function and diffusion tensor and perfusion weighted imaging for high-grade gliomas: A prospective study [file 41598_2019_42321_MOESM1_ESM.pdf]

Supplementary Information for

Effect of radiochemotherapy on the cognitive function and diffusion tensor and perfusion weighted imaging for high-grade gliomas: A prospective study

Yiying Bian[1], Li Meng[2], Jianghua Peng[1], Junfeng Li[1], Rui Wei[1], Lei Huo[3], Huan Yang[4], Ying Wang[5], Jun Fu[1], Liangfang Shen[1], Jidong Hong[1]

Department of [1]Oncology, [2] Radiology, [3]Neurosurgery, [4]Neurology, [5]Pathology, Xiangya Hospital, Central South University, Changsha, 410008 Hunan, PR China. Correspondence and requests for materials should be addressed to J.H. (email: hongjidong1966@126.com)

Table S1 Correlation analysis between parameters of DTI and PWI and dose to brain regions (End of radiotherapy)					
Parameters	Location	Dmax		Dmean	
		r	P	r	P
FA					
	Contralateral frontal lobe	-0.227	0.365	-0.226	0.367
	Contralateral temporal lobe	-0.517	0.028	0.437	0.070
	Contralateral hippocampus	-0.309	0.213	-0.284	0.254
	Ipsilateral hippocampus	-0.427	0.077	-0.339	0.168
	Posterior cingulate gyrus	0.135	0.594	0.148	0.558
	Genu of corpus callosum	-0.093	0.714	-0.160	0.525
	Splenium of corpus callosum	-0.466	0.051	-0.489	0.039
rCBV(ml/100g)					
	Contralateral frontal lobe	0.986	0.006	0.155	0.539
	Contralateral temporal lobe	0.155	0.540	0.281	0.260
	Contralateral hippocampus	0.061	0.809	0.112	0.657
	Ipsilateral hippocampus	0.054	0.830	-0.133	0.598
	Posterior cingulate gyrus	-0.043	0.865	-0.082	0.745
	Genu of corpus callosum	-0.058	0.818	-0.044	0.861
	Splenium of corpus callosum	0.127	0.617	0.144	0.569
rCBF(ml/100g/min)					
	Contralateral frontal lobe	-0.263	0.293	-0.264	0.291
	Contralateral temporal lobe	0.131	0.605	0.096	0.705
	Contralateral hippocampus	0.113	0.656	0.130	0.608

	Ipsilateral hippocampus	−0.132	0.627	−0.121	0.633
	Posterior cingulate gyrus	−0.074	0.771	−0.100	0.694
	Genu of corpus callosum	<0.001	0.999	−0.047	0.852
	Splenium of corpus callosum	−0.246	0.326	−0.169	0.503
MTT(s)					
	Contralateral frontal lobe	0.237	0.344	0.320	0.195
	Contralateral temporal lobe	−0.036	0.887	−0.078	0.757
	Contralateral hippocampus	0.077	0.761	0.177	0.481
	Ipsilateral hippocampus	0.107	0.672	−0.127	0.616
	Posterior cingulate gyrus	−0.230	0.358	−0.179	0.476
	Genu of corpus callosum	−0.267	0.285	−0.200	0.425
	Splenium of corpus callosum	0.305	0.219	0.276	0.268
TTP(s)					
	Contralateral frontal lobe	0.071	0.778	0.203	0.419
	Contralateral temporal lobe	0.083	0.745	−0.008	0.974
	Contralateral hippocampus	0.008	0.975	0.012	0.962
	Ipsilateral hippocampus	0.216	0.390	−0.025	0.922
	Posterior cingulate gyrus	−0.048	0.849	0.017	0.947
	Genu of corpus callosum	0.003	0.991	0.089	0.725
	Splenium of corpus callosum	0.372	0.129	0.377	0.123

r: coefficient of correlation, Dmax: Maximum dose, Dmean: Mean dose, FA: Fractional anisotropy fractional anisotropy, rCBF: regional cerebral blood flow, rCBV: regional cerebral blood volume, MTT: mean transit time, TTP: time to peak

Table S2 Correlation analysis between parameters of DTI and PWI and dose to brain regions (3 months after radiotherapy)

Parameters	Location	Dmax		Dmean	
		r	P	r	P
FA					
	Contralateral frontal lobe	0.150	0.554	0.136	0.590
	Contralateral temporal lobe	0.167	0.507	0.127	0.615
	Contralateral hippocampus	-0.334	0.176	-0.234	0.350
	Ipsilateral hippocampus	-0.683	0.002	-0.532	0.023
	Posterior cingulate gyrus	-0.438	0.069	-0.428	0.077
	Genu of corpus callosum	-0.424	0.080	-0.499	0.035
	Splenium of corpus callosum	-0.374	0.126	-0.375	0.126
rCBV(ml/100g)					
	Contralateral frontal lobe	-0.023	0.928	0.110	0.663
	Contralateral temporal lobe	0.116	0.646	0.124	0.624
	Contralateral hippocampus	-0.132	0.600	-0.038	0.881
	Ipsilateral hippocampus	0.080	0.754	0.112	0.659
	Posterior cingulate gyrus	-0.134	0.597	-0.428	0.077
	Genu of corpus callosum	-0.244	0.329	-0.226	0.366
	Splenium of corpus callosum	-0.384	0.115	-0.316	0.202
rCBF(ml/100g/min)					
	Contralateral frontal lobe	-0.019	0.939	-0.074	0.771
	Contralateral temporal lobe	0.123	0.627	0.017	0.946

	Contralateral hippocampus	-0.181	0.472	-0.072	0.776
	Ipsilateral hippocampus	0.085	0.736	0.090	0.722
	Posterior cingulate gyrus	-0.045	0.860	-0.090	0.721
	Genu of corpus callosum	0.031	0.902	-0.013	0.959
	Splenium of corpus callosum	-0.161	0.523	-0.059	0.816
MTT(s)					
	Contralateral frontal lobe	-0.056	0.826	0.065	0.797
	Contralateral temporal lobe	-0.008	0.976	0.069	0.787
	Contralateral hippocampus	0.078	0.758	0.180	0.476
	Ipsilateral hippocampus	0.014	0.956	0.098	0.697
	Posterior cingulate gyrus	-0.547	0.019	-0.481	0.044
	Genu of corpus callosum	-0.597	0.009	-0.552	0.018
	Splenium of corpus callosum	-0.240	0.338	-0.389	0.111
TTP(s)					
	Contralateral frontal lobe	-0.027	0.915	0.051	0.841
	Contralateral temporal lobe	-0.176	0.485	-0.124	0.624
	Contralateral hippocampus	0.018	0.942	0.011	0.965
	Ipsilateral hippocampus	0.074	0.770	0.206	0.412
	Posterior cingulate gyrus	-0.583	0.011	-0.513	0.030
	Genu of corpus callosum	-0.307	0.215	-0.235	0.348
	Splenium of corpus callosum	-0.419	0.084	-0.559	0.016

r: coefficient of correlation, Dmax: Maximum dose, Dmean: Mean dose, FA: Fractional anisotropy fractional anisotropy, rCBF: regional cerebral blood flow, rCBV: regional cerebral blood volume, MTT: mean transit time, TTP: time to peak

Table S3 Correlation analysis between parameters of DTI and PWI and dose to brain regions(6 months after radiotherapy)

Parameters	Location	Dmax		Dmean	
		r	P	r	P
FA					
	Contralateral frontal lobe	-0.305	0.218	-0.328	0.183
	Contralateral temporal lobe	0.073	0.773	0.077	0.762
	Contralateral hippocampus	0.324	0.190	0.312	0.207
	Ipsilateral hippocampus	-0.477	0.045	-0.397	0.103
	Posterior cingulate gyrus	-0.576	0.012	-0.608	0.007
	Genu of corpus callosum	-0.339	0.168	-0.387	0.113
	Splenium of corpus callosum	-0.222	0.375	-0.257	0.304
rCBV(ml/100g)					
	Contralateral frontal lobe	0.232	0.353	0.329	0.182
	Contralateral temporal lobe	0.137	0.589	0.310	0.210
	Contralateral hippocampus	0.109	0.668	0.136	0.590
	Ipsilateral hippocampus	0.432	0.074	0.286	0.250
	Posterior cingulate gyrus	0.136	0.589	0.107	0.674
	Genu of corpus callosum	-0.058	0.818	0.014	0.957
	Splenium of corpus callosum	-0.042	0.868	-0.028	0.913
rCBF(ml/100g/min)					
	Contralateral frontal lobe	0.046	0.855	-0.022	0.933
	Contralateral temporal lobe	-0.179	0.477	-0.103	0.684

	Contralateral hippocampus	0.195	0.439	0.185	0.461
	Ipsilateral hippocampus	0.282	0.256	0.413	0.089
	Posterior cingulate gyrus	0.034	0.894	0.019	0.942
	Genu of corpus callosum	-0.500	0.034	-0.524	0.026
	Splenium of corpus callosum	0.102	0.687	0.223	0.374
MTT(s)					
	Contralateral frontal lobe	0.142	0.574	0.248	0.321
	Contralateral temporal lobe	0.267	0.285	0.190	0.449
	Contralateral hippocampus	0.084	0.739	0.185	0.463
	Ipsilateral hippocampus	0.580	0.145	0.126	0.619
	Posterior cingulate gyrus	0.034	0.893	0.050	0.844
	Genu of corpus callosum	0.252	0.313	0.282	0.256
	Splenium of corpus callosum	-0.080	0.751	-0.121	0.633
TTP(s)					
	Contralateral frontal lobe	0.256	0.306	0.336	0.173
	Contralateral temporal lobe	-0.576	0.012	0.275	0.269
	Contralateral hippocampus	0.055	0.828	0.036	0.886
	Ipsilateral hippocampus	0.252	0.312	0.007	0.979
	Posterior cingulate gyrus	0.257	0.304	0.304	0.219
	Genu of corpus callosum	0.367	0.134	0.432	0.074
	Splenium of corpus callosum	-0.054	0.833	-0.104	0.681

r: coefficient of correlation, Dmax: Maximum dose, Dmean: Mean dose, FA: Fractional anisotropy fractional anisotropy, rCBF: regional cerebral blood flow, rCBV: regional cerebral blood volume, MTT: mean transit time, TTP: time to peak

Table S4 Correlation analysis between parameters of DTI and PWI and dose to brain regions(9 months after radiotherapy)					
Parameters	Location	Dmax		Dmean	
		r	P	r	P
FA					
	Contralateral frontal lobe	-0.001	0.998	-0.128	0.613
	Contralateral temporal lobe	0.286	0.251	0.299	0.229
	Contralateral hippocampus	0.530	0.150	0.380	0.120
	Ipsilateral hippocampus	-0.339	0.168	-0.195	0.437
	Posterior cingulate gyrus	-0.112	0.657	-0.146	0.563
	Genu of corpus callosum	-0.075	0.767	-0.154	0.541
	Splenium of corpus callosum	-0.237	0.344	-0.219	0.383
rCBV(ml/100g)					
	Contralateral frontal lobe	0.083	0.744	0.303	0.221
	Contralateral temporal lobe	0.081	0.748	0.320	0.195
	Contralateral hippocampus	0.240	0.338	0.276	0.268
	Ipsilateral hippocampus	0.422	0.081	0.384	0.115
	Posterior cingulate gyrus	0.123	0.626	0.057	0.822
	Genu of corpus callosum	-0.117	0.643	-0.050	0.843
	Splenium of corpus callosum	-0.313	0.206	-0.304	0.221
rCBF(ml/100g/min)					
	Contralateral frontal lobe	0.015	0.951	-0.169	0.502
	Contralateral temporal lobe	0.081	0.748	-0.097	0.701

	Contralateral hippocampus	0.271	0.276	0.289	0.245
	Ipsilateral hippocampus	0.120	0.635	0.190	0.451
	Posterior cingulate gyrus	0.220	0.381	0.168	0.506
	Genu of corpus callosum	-0.653	0.003	-0.681	0.002
	Splenium of corpus callosum	-0.002	0.995	0.102	0.687
MTT(s)					
	Contralateral frontal lobe	0.017	0.945	0.194	0.442
	Contralateral temporal lobe	0.202	0.422	0.233	0.353
	Contralateral hippocampus	0.085	0.737	0.185	0.462
	Ipsilateral hippocampus	0.263	0.292	0.178	0.479
	Posterior cingulate gyrus	-0.126	0.618	-0.086	0.734
	Genu of corpus callosum	0.266	0.287	0.319	0.196
	Splenium of corpus callosum	0.228	0.362	0.176	0.484
TTP(s)					
	Contralateral frontal lobe	-0.124	0.624	-0.047	0.852
	Contralateral temporal lobe	0.237	0.343	0.021	0.934
	Contralateral hippocampus	0.028	0.911	0.018	0.943
	Ipsilateral hippocampus	0.225	0.370	0.120	0.635
	Posterior cingulate gyrus	0.085	0.737	0.135	0.592
	Genu of corpus callosum	0.242	0.334	0.306	0.217
	Splenium of corpus callosum	0.146	0.564	0.093	0.714

r: coefficient of correlation, Dmax: Maximum dose, Dmean: Mean dose, FA: Fractional anisotropy fractional anisotropy, rCBF: regional cerebral blood flow, rCBV: regional cerebral blood volume, MTT: mean transit time, TTP: time to peak

Table S5 Correlation analysis between parameters of DTI and PWI and dose to brain regions(12 months after radiotherapy)

Parameters	Location	Dmax		Dmean	
		r	P	r	P
FA					
	Contralateral frontal lobe	-0.590	0.010	-0.504	0.033
	Contralateral temporal lobe	0.157	0.535	0.177	0.483
	Contralateral hippocampus	0.305	0.219	0.365	0.136
	Ipsilateral hippocampus	-0.203	0.420	-0.322	0.192
	Posterior cingulate gyrus	-0.816	<0.001	-0.800	<0.001
	Genu of corpus callosum	-0.395	0.105	-0.450	0.061
	Splenium of corpus callosum	-0.204	0.416	-0.222	0.377
rCBV(ml/100g)					
	Contralateral frontal lobe	-0.237	0.344	0.051	0.840
	Contralateral temporal lobe	0.090	0.722	0.335	0.174
	Contralateral hippocampus	0.790	0.121	0.420	0.083
	Ipsilateral hippocampus	0.296	0.233	0.142	0.574
	Posterior cingulate gyrus	-0.311	0.209	-0.273	0.273
	Genu of corpus callosum	0.047	0.852	0.080	0.752
	Splenium of corpus callosum	-0.227	0.365	-0.292	0.240
rCBF(ml/100g/min)					
	Contralateral frontal lobe	-0.516	0.028	-0.375	0.125
	Contralateral temporal lobe	-0.108	0.669	-0.068	0.788

	Contralateral hippocampus	0.104	0.683	0.159	0.530
	Ipsilateral hippocampus	−0.266	0.286	−0.314	0.205
	Posterior cingulate gyrus	−0.329	0.183	−0.311	0.209
	Genu of corpus callosum	−0.726	0.001	−0.747	<0.001
	Splenium of corpus callosum	0.181	0.473	0.215	0.392
MTT(s)					
	Contralateral frontal lobe	0.058	0.819	0.230	0.358
	Contralateral temporal lobe	0.318	0.198	0.304	0.220
	Contralateral hippocampus	0.085	0.737	0.185	0.462
	Ipsilateral hippocampus	0.296	0.234	0.217	0.387
	Posterior cingulate gyrus	0.157	0.533	0.177	0.482
	Genu of corpus callosum	0.339	0.169	0.800	0.120
	Splenium of corpus callosum	0.209	0.405	0.098	0.698
TTP(s)					
	Contralateral frontal lobe	−0.023	0.927	0.087	0.730
	Contralateral temporal lobe	0.238	0.342	0.109	0.665
	Contralateral hippocampus	−0.005	0.984	0.017	0.948
	Ipsilateral hippocampus	0.327	0.185	0.214	0.393
	Posterior cingulate gyrus	0.305	0.219	0.283	0.255
	Genu of corpus callosum	0.200	0.426	0.223	0.374
	Splenium of corpus callosum	0.152	0.547	0.055	0.830

r: coefficient of correlation, Dmax: Maximum dose, Dmean: Mean dose, FA: Fractional anisotropy fractional anisotropy, rCBF: regional cerebral blood flow, rCBV: regional cerebral blood volume, MTT: mean transit time, TTP: time to peak
